# Extremely Long-Term Glioblastoma Survival (Ten Years): A Possible Role of Immune Modulation From Pre-existing Diseases

**DOI:** 10.7759/cureus.98984

**Published:** 2025-12-11

**Authors:** Diogo Rocha Grade, Tânia Soares, David A João, Tiago Lima, António Marques Baptista

**Affiliations:** 1 Neurosurgery, Unidade Local de Saúde de Gaia e Espinho, Vila Nova de Gaia, PRT; 2 Pathology, Unidade Local de Saúde de Gaia e Espinho, Vila Nova de Gaia, PRT

**Keywords:** crohn disease, glioblastoma, host-pathogen interactions, immunity, long-term survival, syphilis

## Abstract

Glioblastoma (World Health Organization grade 4 astrocytoma) is the most common and aggressive primary brain tumor in adults, with a median survival of 12-15 months despite maximal treatment. Nevertheless, a small subset of patients (approximately 3-5%) survive beyond three years, and fewer than 1% live longer than a decade. Over recent decades, survival outcomes have improved modestly, largely attributable to advancements in surgical techniques, radiotherapy, and chemotherapy. Long-term survival in glioblastoma is thought to arise from a multifactorial interplay between tumor biology, therapeutic response, and host-related characteristics, including immunologic status and systemic conditions that may influence tumor behavior. We report the case of a 54-year-old woman who survived more than ten years following a diagnosis of glioblastoma. Her medical history included Crohn's disease, cigarette smoking, and syphilis treated with penicillin at the age of 21. The patient underwent gross total resection of a left fronto-insular lesion, followed by standard chemoradiotherapy and maintenance temozolomide. Remarkably, ten years after diagnosis, she remains recurrence-free, with a Karnofsky Performance Score of 80 and only mild cognitive impairment attributable to late radiotherapy effects. This case exemplifies an exceptionally favorable long-term outcome in glioblastoma and suggests a potential role for immune modulation associated with chronic inflammatory or infectious conditions in influencing disease course. Further investigation into host-related immune mechanisms may help elucidate factors contributing to durable tumor control in rare long-term survivors.

## Introduction

Glioblastoma (World Health Organization grade 4 astrocytoma) is the most common and aggressive primary malignant brain tumor in adults. Despite maximal treatment consisting of surgical resection, radiotherapy, and temozolomide-based chemotherapy, the median survival remains 12-15 months [1,2]. Disease recurrence is nearly universal, and long-term survival beyond three years occurs in only 3-5% of patients, with fewer than 1% surviving beyond ten years [2,3]. Over recent decades, modest improvements in outcome have been achieved, largely due to refinements in surgical and radiotherapeutic techniques, the introduction of temozolomide, and better integration of molecular diagnostics [4,5]. The extent of resection remains one of the strongest prognostic factors for survival, with near-total or complete resection associated with significantly longer progression-free and overall survival (OS) [6,7].

In addition, comprehensive molecular profiling has revealed considerable biological heterogeneity within glioblastoma, with markers such as MGMT promoter methylation, TERT promoter mutations, and epidermal growth factor receptor (EGFR) amplification influencing prognosis and treatment response [1,8]. Nonetheless, even after accounting for these variables, a small subset of patients, so-called long-term survivors (LTS), demonstrate unexpectedly durable disease control [1,9]. Analyses of such cases have identified no single molecular signature that fully explains survival outliers [8,9]. Instead, evidence suggests a multifactorial process involving both tumor-intrinsic and host-related factors. In particular, immune status and systemic inflammatory conditions have emerged as potential modifiers of disease behavior and treatment efficacy. For example, increased immune cell infiltration, higher expression of antigen-presentation machinery, and favorable tumor-immune microenvironment profiles have been observed in some LTS cohorts [1,9]. Conversely, chronic inflammation, autoimmune disorders, or prior infections may influence immune priming and tumor immunogenicity, potentially modulating disease course. We report the case of a patient who survived more than 10 years following a diagnosis of glioblastoma, analyzing the clinical, pathological, and immunologic context to identify potential determinants of this exceptional outcome. The case highlights the need to further explore host-related immune mechanisms and their contribution to long-term survival in glioblastoma.

## Case presentation

A 54-year-old woman presented in April 2014 with progressive headache and motor aphasia. Her past medical history included Crohn's disease, a history of cigarette smoking, and syphilis, which had been treated with penicillin at the age of 21.

**Figure 1:**
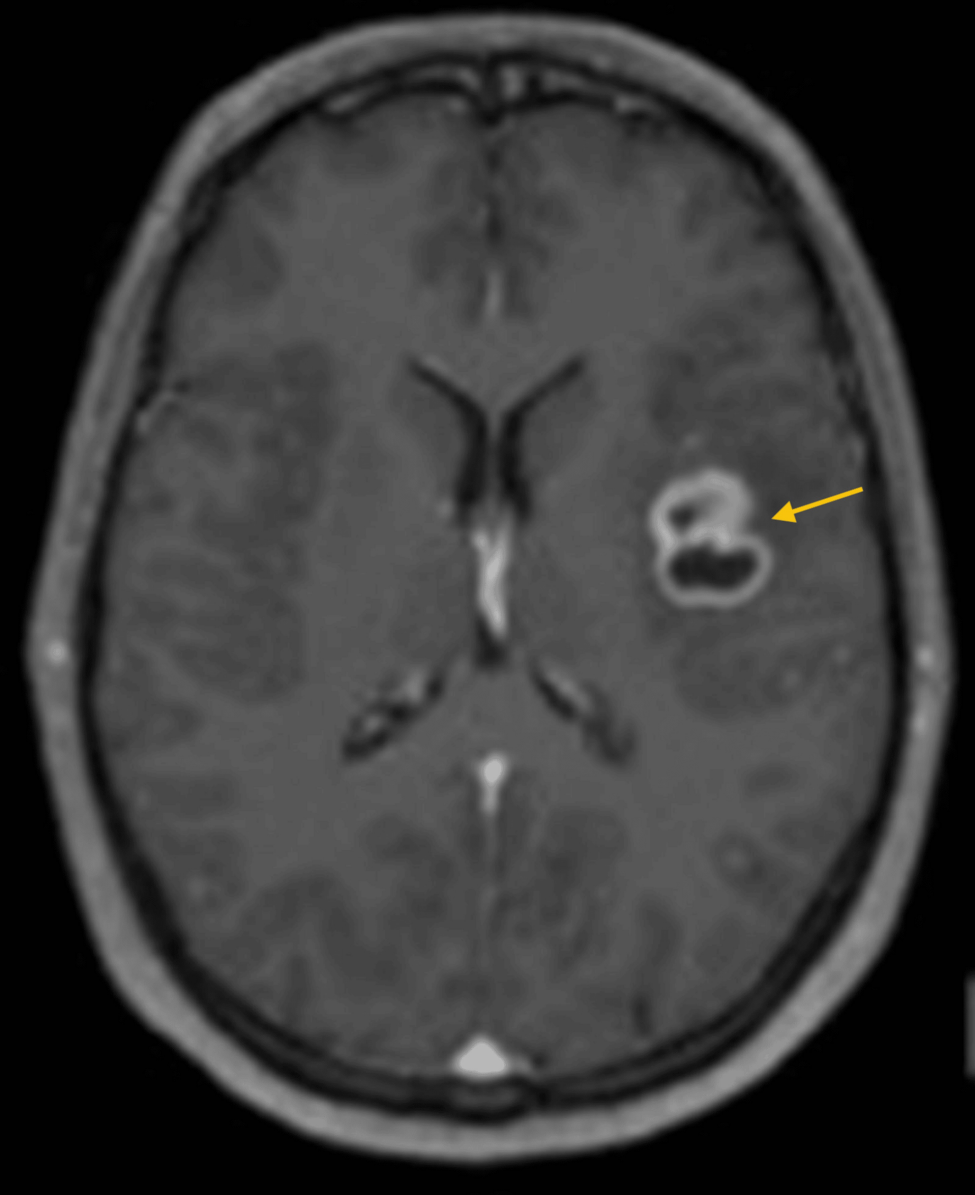
Pre-operative MRI (T1 with gadolinium contrast) The yellow arrow highlights a parenchymal lesion in the left fronto-insular region with contrast enhancement and central necrotic/cystic core, suggestive of a high-grade glioma.

Magnetic resonance imaging (MRI) revealed a single intraparenchymal lesion in the left fronto-insular region, characterized by heterogeneous contrast enhancement and a necrotic/cystic core-findings suggestive of a high-grade glioma (Figure 1).

**Figure 2:**
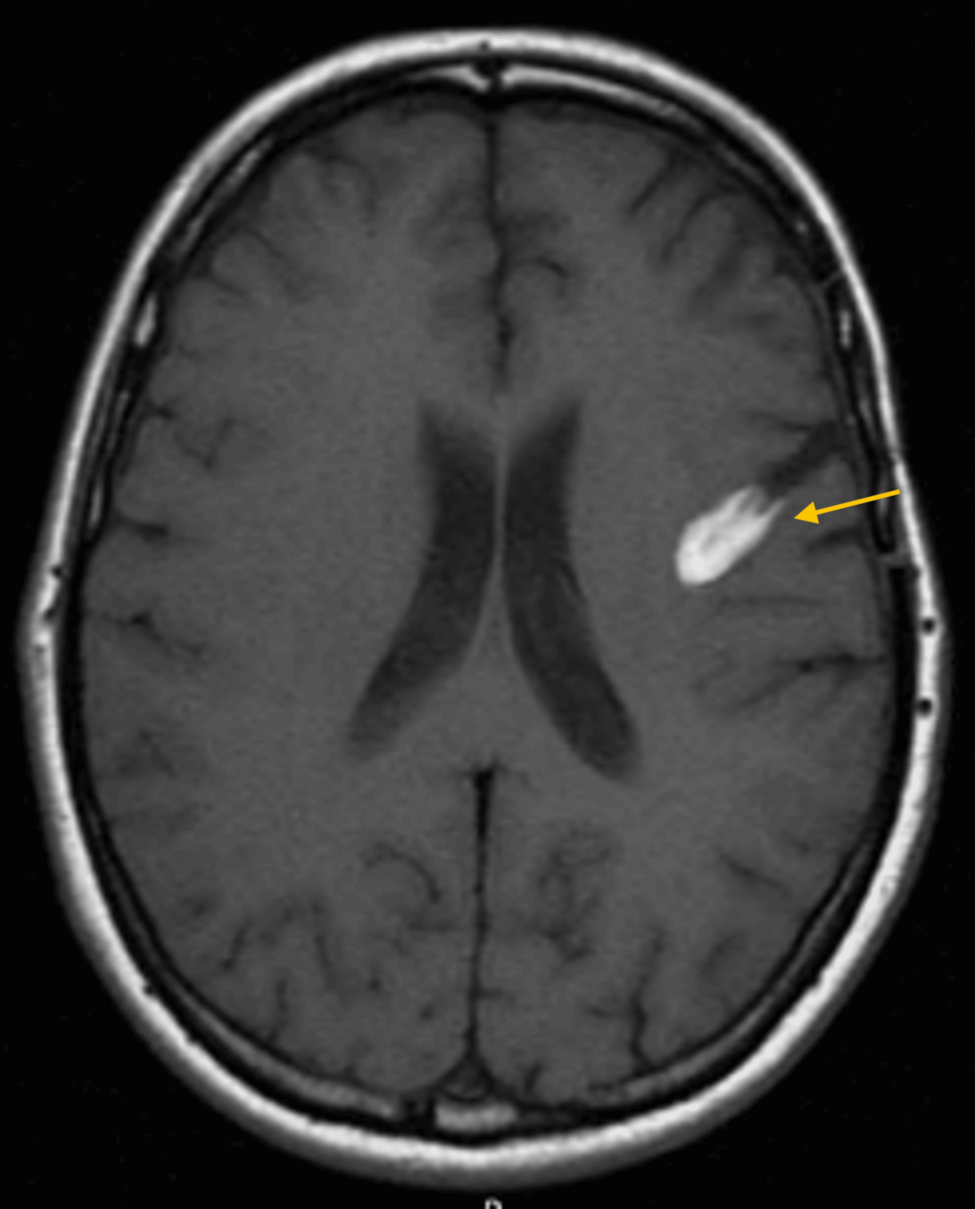
Six-month post-operative MRI The yellow arrow highlights a hyperintense T1-weighted signal at the surgical site, likely related to blood degradation products, without evidence of contrast-enhancing residual or recurrent tumor.

The patient underwent gross total resection of the lesion, with complete postoperative recovery of her language deficits. A follow-up MRI at six months showed a hyperintense T1-weighted signal at the surgical site, likely related to blood degradation products, without evidence of contrast-enhancing residual or recurrent tumor (Figure 2).

**Figure 3:**
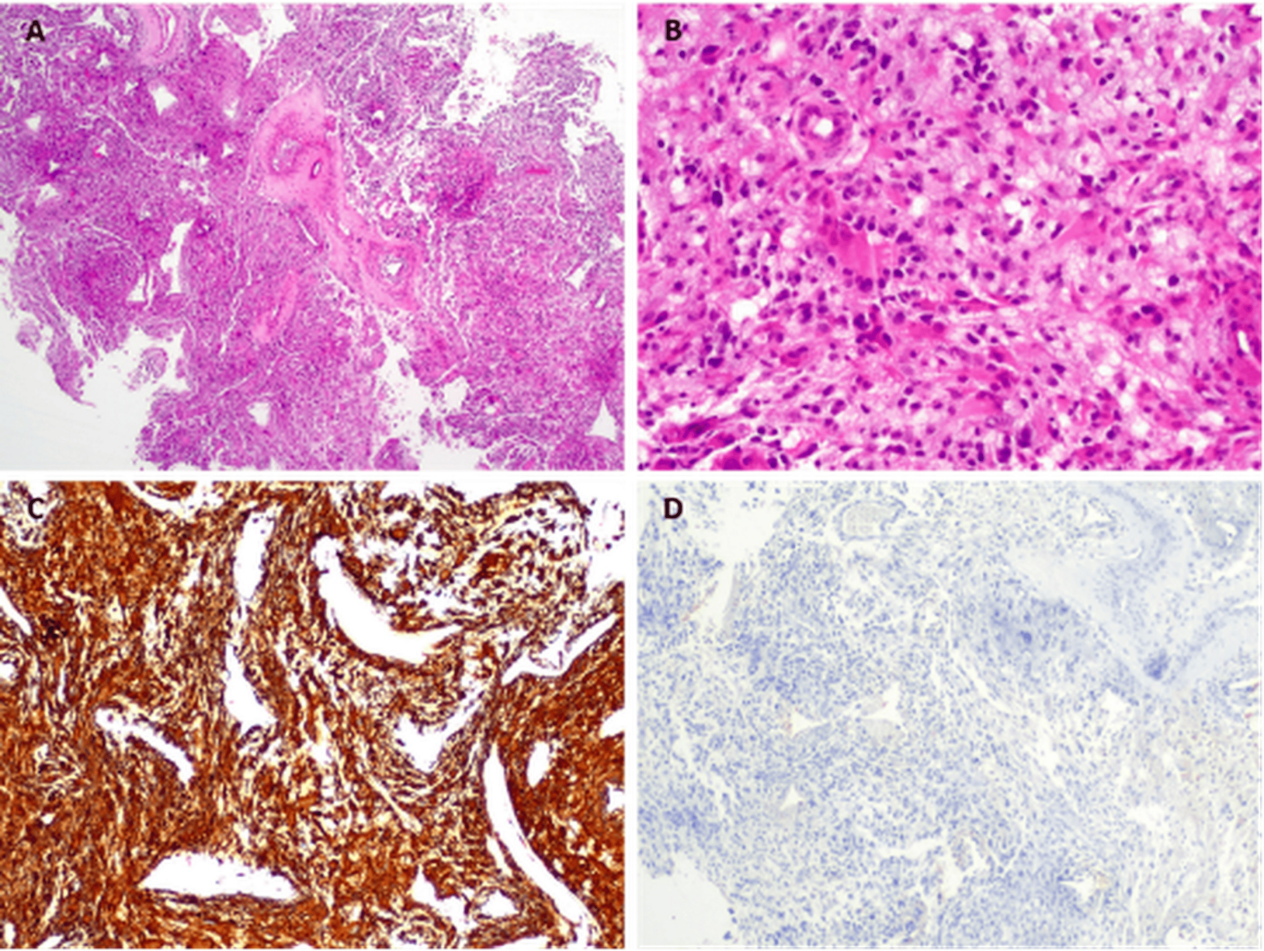
Histopathological examination of brain tumor On Hematoxylin and Eosin staining, a lesion was found, comprised of round to fusiform cells with marked pleomorphism and cytologic atypia. (A and B) There were foci of necrosis, thrombosis, and microvascular proliferation. Immunostaining showed cells with strong positivity for GFAP (C) and no expression of cytokeratins (D). Original magnifications: (A) H&E, x20; (B) H&E, x100; (C) GFAP, x40; (D) CkAE1/AE3, x40

Histopathological examination confirmed the diagnosis of World Health Organization (WHO) grade 4 astrocytoma (glioblastoma) (Figure 3).

At the time of diagnosis, molecular marker testing was not performed, as it was not yet part of the glioma classification criteria.

**Figure 4:**
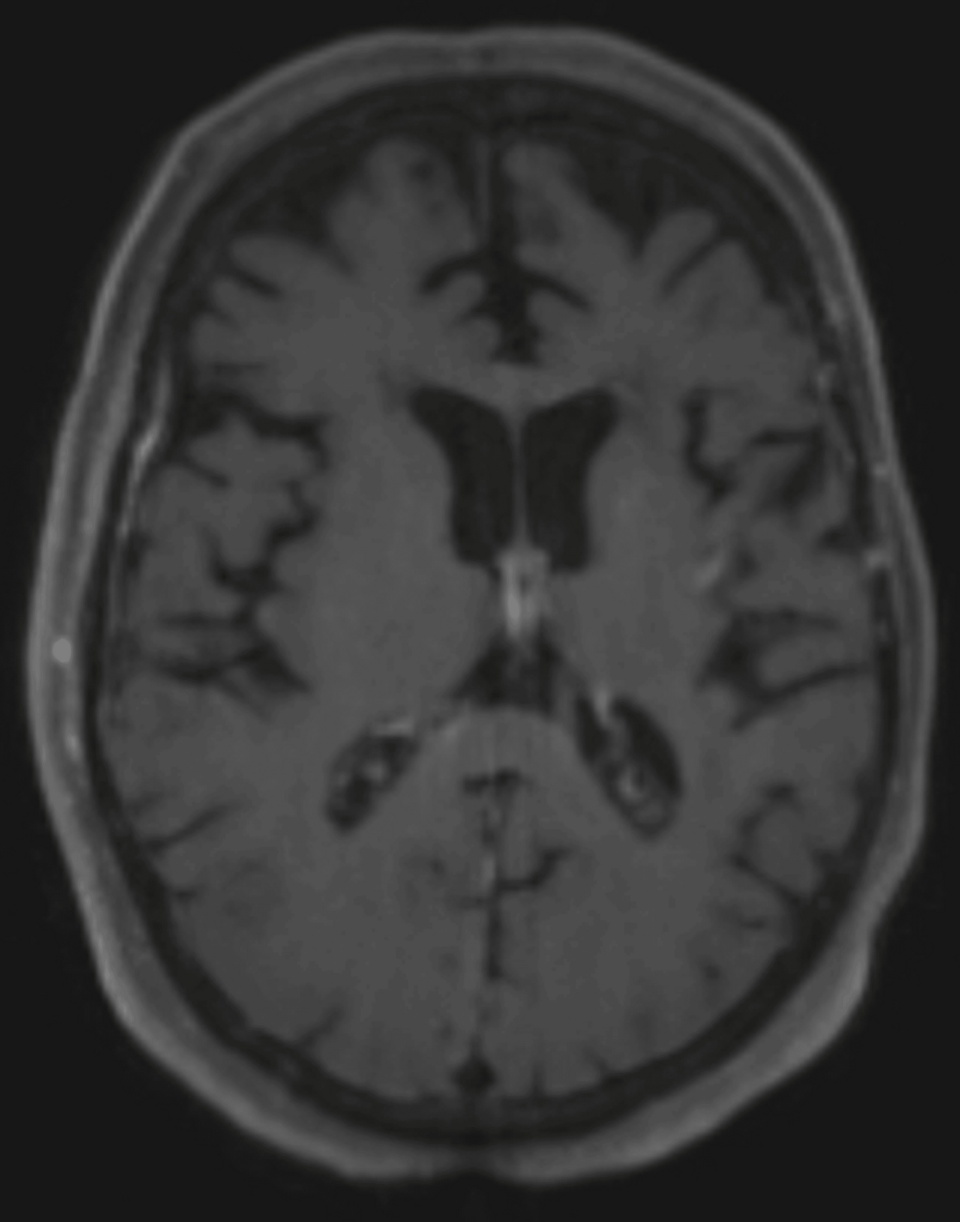
10-year post-operative MRI (T1 with gadolinium contrast) Complete resection without evidence of any lesion.

Standard adjuvant therapy was initiated, consisting of concomitant chemoradiotherapy with temozolomide (75 mg/m²) and fractionated radiotherapy over six weeks. This was followed by maintenance chemotherapy with six cycles of temozolomide (200 mg/m² for five days every 28 days). The patient was followed with regular MRI, and remarkably, ten years after diagnosis, she remains recurrence-free (Figure 4).

She has no focal neurological deficits and maintains a Karnofsky Performance Score (KPS) of 80. Mild cognitive impairment is present, likely attributable to late effects of cranial radiotherapy.

## Discussion

The precise reasons why some patients with glioblastoma (GBM) achieve long-term survival remain uncertain. However, a review of the literature reveals several factors associated with prolonged overall survival, which can be broadly categorized into clinical, treatment-related, and molecular domains.

From a clinical perspective, younger age at diagnosis (typically <60 years) and a high initial Karnofsky Performance Score are among the strongest predictors of long-term survival [1,5]. While gender is generally not considered a significant prognostic factor, some studies report a slight predominance of long-term survivors among female patients [6]. Additionally, longer progression-free survival (PFS) is strongly correlated with improved OS [2,7].

In terms of treatment-related factors, the extent of surgical resection is a well-documented independent predictor of survival. Gross total or subtotal resections are generally associated with better outcomes compared to biopsy alone [5,8]. Nevertheless, the precise threshold of resection necessary to confer this survival benefit is still debated. For example, Sanai et al. reported a significant survival advantage with ≥78% tumor resection [9]. Moreover, approximately half of patients undergo a second craniotomy at the time of first recurrence, which may enhance post-recurrence survival, particularly when gross total resection is achieved, although this remains controversial [10].

While advancements in treatment over recent decades have modestly improved survival, GBM continues to carry a poor prognosis. Accordingly, recent scientific efforts have shifted toward the molecular biology of gliomas in search of reliable prognostic biomarkers. Unfortunately, molecular profiling was not routinely available at our institution at the time of the patient’s initial surgery, which may limit the interpretation of the long-term survival. However, if a second operation had been required more recently, we would have submitted the new specimen for comprehensive molecular profiling. Such an analysis might even help identify a distinct subtype of glioblastoma with a less aggressive biological behavior. Alterations such as isocitrate dehydrogenase (IDH)1/2 mutations, MGMT promoter hypermethylation, absence of EGFR amplification, and TERT promoter mutations have been explored extensively. Yet, cases of long-term survival have been reported even in patients lacking both IDH mutations and MGMT promoter methylation, suggesting that molecular markers alone may not fully account for favorable outcomes [3,4,6]. 

This raises the possibility that host-related factors warrant closer consideration. In our case, comorbid Crohn's disease and a history of syphilis were present, prompting the question of whether these conditions might have contributed to a host-mediated immune response capable of restraining tumor growth.

Although no direct, causal link between Crohn's disease or syphilis and improved GBM survival has been established, both conditions can significantly alter immune system dynamics. Crohn's disease, a chronic autoimmune-mediated inflammatory bowel disease, induces sustained activation of T-cells and macrophages and elevates pro-inflammatory cytokines such as TNF-α, IL-6, and IL-17 [11]. This pro-inflammatory state may enhance the immune system's responsiveness to tumor antigens. Additionally, alterations in gut microbiota associated with Crohn's disease have been shown to influence immunotherapy responses in other cancers and may inadvertently support immune activation in GBM [12].

Similarly, syphilis (caused by *Treponema pallidum*) induces systemic immune activation, characterized by polyclonal B and T cell proliferation, a robust Th1-type response (notably IFN-γ), and recruitment of cytotoxic CD8+ T cells and macrophages [13,14]. Such responses could contribute to a state of "trained immunity," in which chronic infections prime innate immune cells such as monocytes and microglia for heightened reactivity to subsequent stimuli [15,16]. Furthermore, cross-reactive T cell priming may occur, whereby T cell clones generated in response to *T. pallidum* may also recognize tumor-associated antigens, enhancing immune surveillance in the central nervous system (CNS) [17]. Table 1 summarizes the currently recognized prognostic factors for glioblastoma and includes Crohn's disease and prior syphilis infection as speculative, host-related variables. Their inclusion reflects the hypothesis that these immune-modulating conditions may influence tumor behavior, while acknowledging that definitive evidence is lacking and warrants further investigation.

**Table 1:** Summary of recognized GBM prognostic factors and corresponding findings in the present patient GBM - glioblastoma; KPS - Karnofsky Performance Score; PFS - progression-free survival; OS - overall survival; IDH - isocitrate dehydrogenase; EGFR - epidermal growth factor receptor

Prognostic factor		Favorable prognostic pattern	Patient status	Alignment
Clinical	Age	<60 years	54	Yes
	KPS	High (≥80–90)	100%	Yes
	Sex	Female (slight trend)	Female	Yes
	PFS	Longer PFS predicts OS	Very long PFS (>10 years)	Yes
Treatment-related	Extent of resection	Gross total/subtotal resection	Gross total resection	Yes
	Adjuvant chemoradiotherapy	Significantly improves prognosis	Accomplished	Yes
Molecular	IDH Mutation	Mutant = favorable	Unknown	Unknown
	MGMT methylation	Methylated = favorable	Unknown	Unknown
	EGFR amplification	Absence = favorable	Unknown	Unknown
	TERT mutation	Variable, subtype‑dependent	Unknown	Unknown
Host-derived	Crohn's Disease	Not yet a recognized prognostic factor; theoretical immune activation	Present	Potential immune benefit
	History of syphilis	Not yet a recognized prognostic factor; may induce trained immunity	Present	Potential immune benefit

Summarizing the concept, it is conceivable that a glioblastoma patient with both Crohn's disease and a history of syphilis may benefit from a chronically activated immune system that is more adept at recognizing and combating tumor cells. Trained innate immunity and adaptive immune memory responses could underlie enhanced tumor control and contribute to prolonged survival [12,14,15].

## Conclusions

Although direct evidence linking Crohn's disease or syphilis to improved GBM outcomes is currently limited, emerging research suggests that these conditions may modulate the immune system in ways that could theoretically influence GBM progression. Specifically, the chronic immune activation associated with both Crohn's disease and syphilis may enhance immune surveillance, potentially increasing the detection and elimination of tumor cells. Additionally, these immune alterations could modulate the tumor microenvironment through changes in microglial activation and cytokine expression, creating conditions that are less favorable for tumor growth. However, it is important to emphasize that these hypotheses remain speculative, and further research is necessary to clarify any potential causal relationships.
